# Clouded Issues for *PHACTR1*

**DOI:** 10.3390/ijms16059770

**Published:** 2015-04-29

**Authors:** Philipp G. Sand

**Affiliations:** 1 Department of Psychiatry, University of Regensburg, 93042 Regensburg, Germany; E-Mail: philipp.sand@ukr.de; Tel.: +49-941-941-8041; Fax: +49-941-941-1235; 2 Mental Health Services, Danuvius Klinik GmbH, 85049 Ingolstadt, Germany

I have read with interest the recent paper by Han and coworkers [1] on the putative effects of a *PHACTR1* variant in the context of coronary artery disease. The authors conclude to a significant risk-enhancing role of rs12526453 on the grounds of 19 earlier case-control studies. Key issues, however, remain unanswered. It is not clear from the 18-page article whether the claim refers to a genotypic or an allelic risk model, let alone which genotype, or allele, is actually considered risk-enhancing. The calculations of Han and colleagues are also a concern in that the data extracted from the literature deviate markedly from the original findings. Thus, cases and controls have been muddled [2], fictitious cases and controls have been introduced (e.g., from a genome-wide association study phase that did not include rs12526453 [3]), odds ratios have been altered [4,5] and quantitative traits have been arbitrarily dichotomized *post hoc* to fit in a case-control design [6]. Other inconsistencies include discrepant measures of effect size (e.g., Table 5 and Figure 3 [1]), contradictory numbers in article retrieval (see Figure 1 [1]), missing data points (see Figure 5 [1]) and utterly erratic referencing.

For their own case-control study, the authors have chosen a common age cut-off for defining high-risk subpopulations despite ample evidence that coronary artery disease manifests seven to 10 years later in women than it does in men [7]. Overall, some 48 individual tests were used without correcting for multiple comparisons. Unordered Chi-square tests should be replaced by the Armitage trend test for the examination of differences in genotype counts. Most importantly, genetic exposure to rs12526453 is currently equivocal owing to insufficient information. Unless the DNA strand examined is specified, the “C” allele gets mistaken for the “G” allele and *vice versa*, rendering genotype results meaningless. Those earlier association studies that fail to provide this information cannot be pooled to augment statistical power as has been done. I propose that the authors reperform their analyses so that an accurate estimate may be obtained of the true risk conferred by *PHACTR1* on coronary artery disease.
